# A study on the status of myopia and pre-myopia among primary school students in different regions of Shaanxi Province, China

**DOI:** 10.3389/fped.2025.1596389

**Published:** 2025-06-12

**Authors:** Dong Wei, Li Yangbing, Liu Chengfei, Zhang Ye, Wang Zihao, Li Yanying, He Xiaokang, Fu Kunlin, Du Zhaojiang

**Affiliations:** ^1^ Department of Ophthalmology, Xi'an Central Hospital, Xi'an, China; ^2^ Department of Clinical Medicine, Yan'an University, Yan'an, Shaanxi, China; ^3^ Department of Urology, Xi'an Daxing Hospital, Xi'an, Shaanxi, China; ^4^ Department of Ophthalmology, Maternal and Child Health Care Hospital of Dingbian County, Yulin, Shaanxi, China

**Keywords:** myopia epidemiology, pre-myopia, school-age children, geographic disparity, refractive error, China

## Abstract

**Objective:**

This cross-sectional study aimed to investigate the geographic disparities in myopia and pre-myopia prevalence among elementary school students across three distinct regions of Shaanxi Province (southern Hanzhong, Guanzhong, and northern Yulin) to inform region-specific myopia control strategies.

**Methods:**

From March to May 2024, we employed multistage cluster sampling to recruit 8,207 eligible students (2,724 southern Shaanxi, 2,761 Guanzhong, 2,722 northern Shaanxi) from 12 randomly selected primary schools. Comprehensive ophthalmic examinations including uncorrected visual acuity and non-cycloplegic autorefraction were conducted. Continuous variables were expressed as mean ± standard deviation, while categorical variables were analyzed using chi-square tests.

**Results:**

Age-standardized myopia prevalence was highest in northern Shaanxi (48.02%), followed by central Shaanxi/Guanzhong (42.96%) and southern Shaanxi (30.43%). Gender disparities persisted across all regions, with female students exhibiting significantly elevated myopia rates (southern Shaanxi: 34.00% vs. 26.91%; Guanzhong: 48.02% vs. 37.99%; northern Shaanxi: 52.54% vs. 44.13%; *P* < 0.05 for all comparisons). Pre-myopia prevalence displayed an inverse geographic pattern (southern Shaanxi: 40.60% > Guanzhong: 34.19% > northern Shaanxi: 33.73%; *χ*^2^ = 185.3, *P* < 0.001), with male students consistently showing higher pre-myopia detection rates than females (southern Shaanxi: 42.45% vs. 38.73%; Guanzhong 38.28% vs. 30.01%; northern Shaanxi: 37.64% vs. 29.17%; *P* < 0.05). A marked grade-level progression was observed, with myopia prevalence increasing annually while pre-myopia rates declined significantly.

**Conclusion:**

Our findings reveal a north–south gradient in ocular health outcomes, with northern Shaanxi demonstrating concerningly high myopia prevalence coupled with reduced pre-myopia detection rates. The persistent female predominance in myopia burden and early detection gaps underscores the need for gender-sensitive interventions. The observed progression patterns suggest critical windows for prevention, advocating for: (1) Preschool-initiated vision protection programs, (2) Establishment of digital refractive registries for high-risk cohorts, and (3) Implementation of regionally tailored myopia control protocols prioritizing northern districts.

## Introduction

Myopia has emerged as a global public health concern, with its prevalence escalating at an alarming rate worldwide. As the leading cause of visual impairment, myopia not only compromises individuals' quality of life but also poses significant ocular health risks. Of particular concern is high myopia, which induces axial elongation of the eye, thereby dramatically increasing the risk of sight-threatening complications including retinal detachment, retinal tears, and myopic macular degeneration (1). The socioeconomic impact of myopia is equally substantial, with China bearing an enormous economic burden. Recent epidemiological studies have revealed that the annual expenditure for myopia treatment and prevention in China reaches 9.4 billion in productivity losses due to severe visual impairment, culminating in a staggering total annual economic burden of $26.3 billion (2). These compelling statistics underscore the critical importance of implementing effective myopia prevention strategies. Despite the growing body of research on myopia epidemiology, there remains a notable gap in comparative analyses of myopia prevalence across different regions of Shaanxi Province, particularly between Guanzhong, southern Shaanxi, and northern Shaanxi. To address this knowledge gap and enhance myopia prevention efforts, we have implemented an innovative “medical-educational-family” collaborative model for school-based vision screening. This comprehensive approach enables systematic monitoring of visual acuity and refractive status among primary and secondary school students through biannual examinations conducted in both spring and fall semesters. The data collected through this initiative provide valuable insights and scientific evidence to inform and optimize myopia prevention and control strategies in the region.

## Methods

### Study participants

**Figure 1:**
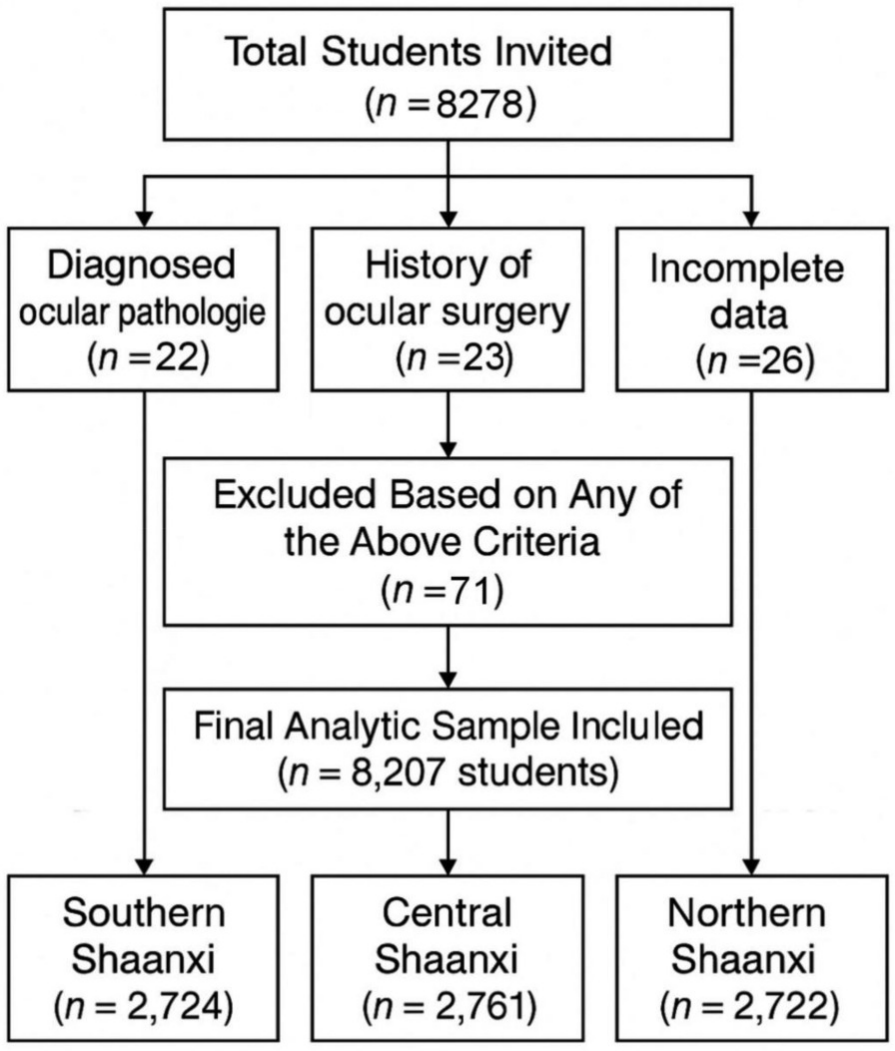
A flowchart showing the students affected by the various exclusion criteria.

**Figure 2:**
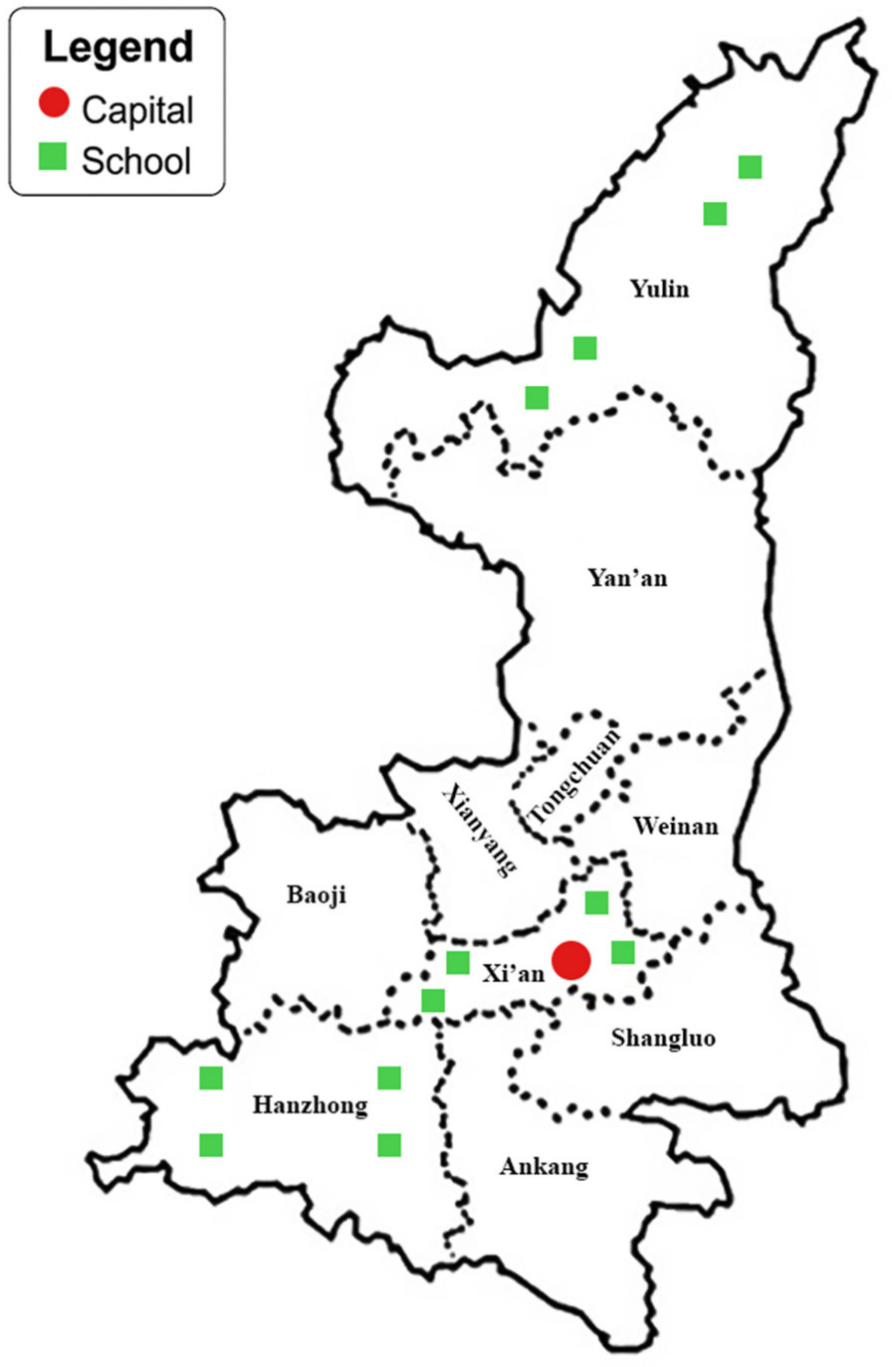
A map illustrating the Shaanxi region, along with the approximate location of the schools involved.

This cross-sectional study was conducted between March and May 2024 across three distinct geographical regions of Shaanxi Province, China: Hanzhong (Southern Shaanxi), Xi'an (Guanzhong Plain), and Yulin (Northern Shaanxi). Using a multistage stratified random sampling approach, we first compiled a comprehensive list of all elementary schools within each region—433 in Southern Shaanxi, 1,169 in Guanzhong, and 312 in Northern Shaanxi. From these, 4 schools per region (totaling 12 schools) were randomly selected as primary sampling units. In each school, the three classes per level (Grades 1–6) were selected using a simple random sampling method from all available parallel classes at each grade level, ensuring representativeness while maintaining logistical feasibility. All enrolled students underwent standardized visual acuity testing and non-cycloplegic autorefraction assessments. Exclusion criteria included: (1) diagnosed ocular pathologies (amblyopia, strabismus, congenital glaucoma, or congenital cataracts); (2) history of ocular surgery; (3) incomplete examination data. After applying these exclusion criteria, the final analytic sample comprised 8,207 participants, distributed as follows: 2,724 from Southern Shaanxi, 2,761 from Guanzhong, and 2,722 from Northern Shaanxi. A flowchart showing the students affected by the various exclusion criteria is presented in Figure 1. A map illustrating the Shaanxi region, along with the approximate location of the schools involved can be found in Figure 2.

### Ethical considerations

The study protocol was approved by the Medical Ethics Committee of Xi'an central hospital (Approval No. LW-2024-027) and conducted in accordance with the Declaration of Helsinki. Written informed consent was obtained from all participants' legal guardians prior to data collection.

### Study examinations

Visual acuity assessment was performed by certified ophthalmologists using the standardized E-Chart logarithmic visual acuity system (GB 11533-2011). Participants were positioned at 5 meters from the chart under ambient illumination of 300–500 lux. Monocular measurements followed standardized protocols, with right eyes examined prior to left eyes, recording uncorrected visual acuity (UCVA) values.

Refractive measurements were conducted without cycloplegia using the Topcon KP-800 automated refractometer (Topcon Corporation, Tokyo, Japan), operated by licensed optometrists. Three consecutive measurements were obtained for each eye (right eye first) to determine spherical power, cylindrical power, axial orientation, and spherical equivalent. The final refractive values represented the arithmetic mean of triplicate measurements. All data were automatically transmitted via secure cloud-based interface to the Shaanxi Provincial Student Vision Surveillance Database.

To ensure data integrity, a multi-level quality control system was implemented: (1) Daily calibration of instruments using NIST-traceable reference standards; (2) Random duplicate testing of 5% participants with <5% inter-test discrepancy threshold for spherical equivalent (95% limits of agreement within ±0.25 D); (3) Automated outlier detection algorithms flagging measurements beyond ±3SD from cohort means for manual verification.

In addition to student vision assessments, a structured parental questionnaire was distributed to the legal guardians of all participants to assess knowledge, attitudes, and practices (KAP) related to myopia prevention. The questionnaire was developed based on validated KAP frameworks and reviewed by ophthalmology and public health experts for content validity. It included 15 items covering awareness of myopia risk factors, understanding of preventive strategies (e.g., outdoor activity, screen time management), and engagement with school-based vision programs. Questionnaires were distributed via school communication channels and collected within one week. A total of 7,894 completed responses (96.2% response rate) were included in the final analysis.

### Definition

Myopia classification was established according to internationally recognized criteria (3–5). Screening myopia was characterized by a spherical equivalent (SE) ≤ −0.50 diopters (D) in at least one eye accompanied by uncorrected visual acuity <5.0 on standard logarithmic charts (3). The severity of refractive error was stratified as follows: low myopia (−3.00 D < SE ≤ −0.50 D), moderate myopia (−6.00 D < SE ≤ −3.00 D), and high myopia (SE ≤ −6.00 D) (4). Pre-myopia status was identified as eyes demonstrating borderline refractive values within the range of −0.50 D < SE ≤  + 0.75 D, in accordance with the WHO definition for individuals at risk of myopia development (5).

### Statistical analysis

All statistical analyses were performed using SPSS (Version 27.0; IBM Corp.) and Microsoft Excel (Office 2019) software. Continuous variables with approximately normal distribution were expressed as mean ± standard deviation (SD), as confirmed by Shapiro–Wilk normality testing. Refractive error measurements demonstrated symmetrical interocular agreement across all age groups (Mann–Whitney *U* test, *P* > 0.05 for right vs. left eye spherical equivalent comparisons). Consequently, right-eye data were selected as representative values for subsequent analyses to maintain methodological consistency. Categorical variables were reported as frequency distributions with percentages. Intergroup comparisons of categorical outcomes were evaluated using Pearson's chi-square test. A two-tailed *P* value <0.05 was established as the threshold for statistical significance.

## Results

### Geographical distribution of refractive status among primary school students

The cross-sectional study enrolled 8,207 primary school students stratified into three geographical regions: Southern Shaanxi (*n* = 2,724; 33.2%), Guanzhong (*n* = 2,761; 33.6%), and Northern Shaanxi (*n* = 2,722; 33.2%). The cohort had a mean age of 8.78 ± 1.52 years (range: 6–12 years), with balanced gender distribution across regions (*χ*^2^ = 1.85, *P* = 0.39).

As detailed in Table 1, spherical equivalent (SE) progression demonstrated significant grade-level dependence in all regions. Southern Shaanxi exhibited the most gradual myopic shift, with mean SE values progressing from −0.43 ± 0.78 D in Grade 1 to −1.32 ± 1.28 D in Grade 6. The Guanzhong region showed accelerated refractive changes, advancing from −0.44 ± 0.90 D to −2.25 ± 1.95 D across the same grade span. Northern Shaanxi displayed intermediate progression rates, ranging from −0.38 ± 1.39 D (Grade 1) to −2.18 ± 2.22 D (Grade 6). Regional comparison of progression slopes revealed statistically significant differences (ANCOVA adjusted for age, *F* = 18.34, *P* < 0.05), with Guanzhong demonstrating the steepest annual myopic progression rate (−0.30 D/year, 95% CI: −0.34 to −0.26).

**Table 1:** Geographical distribution of refractive Status Among primary school students.

Grade	*N* (%)			D (mean ± SD)		
	Southern Shaanxi	Guan zhong	Northern Shaanxi	Southern Shaanxi	Guan Zhong	Northern Shaanxi
Grade 1	472 (17.3)	458 (16.6)	442 (16.2)	−0.43 ± 0.78	−0.44 ± 0.90	−0.38 ± 1.39
Grade 2	400 (14.7)	489 (17.7)	442 (16.2)	−0.44 ± 0.64	−0.55 ± 1.30	−0.42 ± 1.37
Grade 3	411 (15.1)	471 (17.1)	483 (17.7)	−0.59 ± 0.92	−0.97 ± 1.37	−0.91 ± 1.64
Grade 4	427 (15.7)	447 (16.2)	458 (16.8)	−0.67 ± 1.04	−1.32 ± 1.55	−1.36 ± 1.70
Grade 5	479 (17.6)	424 (15.4)	448 (16.5)	−1.05 ± 1.05	−1.75 ± 1.59	−1.76 ± 1.96
Grade 6	535 (19.6)	472 (17.0)	449 (16.6)	−1.32 ± 1.28	−2.25 ± 1.95	−2.18 ± 2.22

### Geographical and gender disparities in myopia prevalence Among shaanxi primary school students

**Figure 3:**
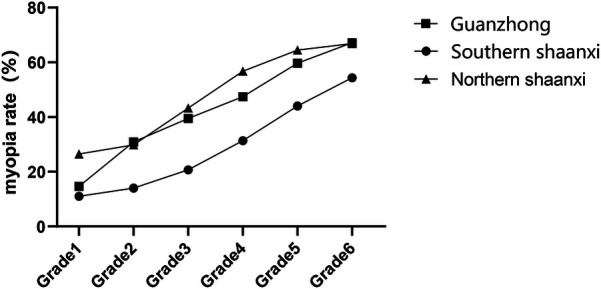
Myopia rates among primary school students in southern Shaanxi, Guanzhong and northern Shaanxi.

The age-standardized myopia prevalence demonstrated significant geographical variation across Shaanxi Province (*χ*^2^ = 185.3, *P* < 0.05). Southern Shaanxi exhibited the lowest prevalence (30.43%; 95% CI: 28.7–32.2), followed by Guanzhong (42.96%; 95% CI: 41.1–44.8) and Northern Shaanxi (48.02%; 95% CI: 46.2–49.8). Grade-stratified analysis revealed a consistent positive correlation between educational progression and myopia risk (Southern *χ*^2^ = 340.7, Guanzhong *χ*^2^ = 346.2, Northern *χ*^2^ = 271.4; all *P* < 0.05, Figure 3).

### Severity distribution patterns

Low myopia (−3.00 D < SE ≤ −0.50 D) predominated across all regions, accounting for 70%–86% of cases. However, Northern Shaanxi demonstrated significantly higher age-adjusted prevalence of high myopia (SE ≤ −6.00 D) compared to other regions (4.06% vs. 2.53% in Guanzhong vs. 0.12% in Southern; *χ*^2^ = 28.4, *P* < 0.05).

### Gender-specific epidemiology

Consistent female predominance was observed across all regions:

Southern Shaanxi: 34.00% (F) vs. 26.91% (M); *χ*^2^ = 16.1, *P* < 0.05

Guanzhong: 48.02% (F) vs. 37.99% (M); *χ*^2^ = 28.3, *P* < 0.05

Northern Shaanxi: 52.54% (F) vs. 44.13% (M); *χ*^2^ = 19.2, *P* < 0.05

The regional hierarchy (Northern Shaanxi > Guanzhong > Southern Shaanxi) persisted within gender subgroups (Male: *χ*^2^ = 92.4; Female: *χ*^2^ = 100.1; both *P* < 0.001), with Northern Shaanxi females exhibiting the highest overall prevalence (Tables 2–5).

**Table 2:** Myopia among primary school students in Southern Shaanxi.

Grade	Male			Female			Total		
	*n*	myopia (*n*)	(%)	*n*	myopia (*n*)	(%)	*n*	myopia (*n*)	(%)
Grade 1	228	25	8.77	244	27	11.07	472	52	11.02
Grade 2	217	30	13.82	183	26	14.21	400	56	14.00
Grade 3	194	37	19.07	217	48	22.12	411	85	20.68
Gade 4	222	61	27.48	205	73	35.61	427	134	31.38
Grade 5	234	91	38.89	245	120	48.98	479	211	44.05
Grade 6	276	125	45.29	259	166	64.09	535	291	54.39
Total	1,371	369	26.91	1,353	460	34.00	2,724	829	30.43

**Table 3:** Myopia among primary school students in Guanzhong.

Grade	Male			Female			Total		
	*n*	myopia (*n*)	(%)	*n*	myopia (*n*)	(%)	*n*	myopia (*n*)	(%)
Grade 1	236	27	11.44	222	40	18.02	458	67	14.63
Grade 2	263	69	26.24	226	82	36.28	489	151	30.88
Grade 3	260	97	37.31	211	89	42.18	471	186	39.49
Grade 4	219	98	44.75	228	114	50.00	447	212	47.43
Grade 5	198	109	55.05	226	144	63.72	424	253	59.67
Grade 6	219	130	59.36	253	187	73.91	472	317	67.16
Total	1,395	530	37.99	1,366	656	48.02	2,761	1,186	42.96

**Table 4:** Myopia among primary school students in Northern Shaanxi.

Grade	Male			Female			Total		
	*n*	myopia (*n*)	(%)	*n*	myopia (*n*)	(%)	*n*	myopia (*n*)	(%)
Grade 1	246	64	26.02	196	53	27.04	442	117	26.47
Grade 2	242	68	28.10	200	64	32.00	442	132	29.86
Grade 3	243	97	39.92	240	112	46.67	483	209	43.27
Grade 4	231	117	50.65	227	143	62.70	458	260	56.77
Grade 5	256	152	59.38	192	137	71.35	448	289	64.51
Grade 6	246	148	60.16	203	152	74.88	449	300	66.82
Total	1,464	646	44.13	1,258	661	52.54	2,722	1,307	48.02

**Table 5:** Geographical disparities in myopia severity among Shaanxi primary school students.

Region	*n*	myopia (*n*)	Degree of myopia		
			Low	Moderate	High
Southern Shaanxi	2,724	829 (30.43)	712 (85.89)	116 (13.99)	1 (0.12)
Guanzhong	2,761	1,186 (42.96)	831 (70.07)	325 (27.40)	30 (2.53)
Northern Shaanxi	2,722	1,307 (48.02)	917 (70.16)	337 (25.78)	53(4.06)

### Geographical and gender variations in pre-myopia prevalence

**Figure 4:**
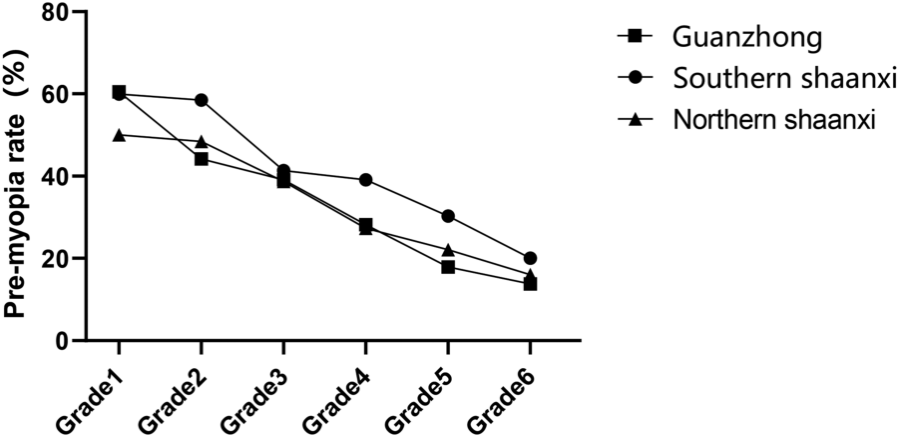
Pre-myopia rates among primary school students in southern Shaanxi, Guanzhong and northern Shaanxi.

The age-adjusted pre-myopia prevalence exhibited inverse geographical distribution compared to established myopia patterns (*χ*^2^ = 185.3, *P* < 0.05). Southern Shaanxi demonstrated the highest prevalence (40.60%), followed by Guanzhong (34.19%) and Northern Shaanxi (33.73%). A significant negative correlation with grade progression was observed across all regions (Southern Shaanxi *χ*^2^ = 242.3, Guanzhong *χ*^2^ = 311.8, Northern Shaanxi *χ*^2^ = 198.9; all *P* < 0.05) (Figure 4).

### Gender disparity patterns

Consistent male predominance was identified in pre-myopia distribution:

Southern Shaanxi: 38.73% (F) vs. 42.45% (M); *χ*^2^ = 3.9, *P* < 0.05

Guanzhong: 30.01% (F) vs. 38.28% (M); *χ*^2^ = 21.0, *P* < 0.05

Northern Shaanxi: 29.17% (F) vs. 37.64% (M); *χ*^2^ = 21.7, *P* < 0.05

The regional hierarchy (Southern Shaanxi > Guanzhong > Northern Shaanxi) remained significant within gender subgroups (Males: *χ*^2^ = 8.0; Females: *χ*^2^ = 33.9; all *P* < 0.05) (Tables 6–8).

**Table 6:** Pre-myopia among primary school students in Southern Shaanxi.

Grade	Male			Female			Total		
	*n*	myopia (*n*)	(%)	*n*	myopia (*n*)	(%)	*n*	myopia (*n*)	(%)
Grade 1	228	138	60.52	244	145	59.43	472	283	59.96
Grade 2	217	128	58.99	183	106	57.92	400	234	58.50
Grade 3	194	81	41.75	217	89	41.01	411	170	41.36
Grade 4	222	92	41.44	205	75	36.59	427	167	39.11
Grade 5	234	73	31.20	245	72	29.39	479	145	30.27
Grade 6	276	70	25.36	259	37	14.29	535	107	20.00
Total	1,371	582	42.45	1,353	524	38.73	2,724	1,106	40.60

**Table 7:** Pre-myopia among primary school students in Guanzhong.

Grade	Male			Female			Total		
	*n*	myopia (*n*)	(%)	*n*	myopia (*n*)	(%)	*n*	myopia (*n*)	(%)
Grade 1	236	148	62.71	222	129	58.11	458	277	60.48
Grade 2	263	124	47.15	226	92	40.71	489	216	44.17
Grade 3	260	107	41.15	211	77	36.49	471	184	39.07
Grade 4	219	68	31.05	228	58	25.44	447	126	28.19
Grade 5	198	43	21.72	226	33	14.60	424	76	17.92
Grade 6	219	44	20.09	253	21	8.30	472	65	13.77
Total	1,395	534	38.28	1,366	410	30.01	2,761	944	34.19

**Table 8:** Pre-myopia among primary school students in Northern Shaanxi.

Grade	Male			Female			Total		
	*n*	myopia (*n*)	(%)	*n*	myopia (*n*)	(%)	*n*	myopia (*n*)	(%)
Grade 1	246	126	51.22	196	95	48.47	442	221	50.00
Grade 2	242	123	50.81	200	91	45.50	442	214	48.42
Grade 3	243	106	43.62	240	81	33.75	483	187	38.72
Grade 4	231	76	32.90	227	49	21.59	458	125	27.29
Grade 5	256	65	25.39	192	34	17.71	448	99	22.10
Grade 6	246	55	22.36	203	17	8.37	449	72	16.04
Total	1,464	551	37.64	1,258	367	29.17	2,722	918	33.73

Our study reveals significant regional disparities in myopia prevalence among primary school students across three distinct geographical divisions of Shaanxi Province: Southern Shaanxi, Guanzhong, and Northern Shaanxi. The hierarchical pattern of myopia prevalence—Northern Shaanxi > Guanzhong > Southern Shaanxi—persists even when controlling for grade-level variations, with Northern regions additionally demonstrating greater severity of refractive errors.

This geographical pattern likely reflects the complex interplay between educational stressors and preventive interventions. Students in Guanzhong reported significantly longer near-work duration and higher participation in after-school tutoring, yet demonstrated better myopia control outcomes. Parental questionnaires revealed that 71.2% of Guanzhong caregivers could accurately describe ≥3 myopia prevention strategies, compared to 53.1% in Northern regions (*χ*^2^ = 18.43, *p* < 0.001). These findings corroborate previous evidence that caregiver health literacy significantly mediates myopia progression through optimized behavioral interventions (6, 7).

The relatively favorable ocular outcomes in Southern Shaanxi may be multifactorial: (1) traditional agricultural lifestyles potentially provide more cycloplegic outdoor exposure; (2) lower population density reduces visual crowding effects. However, our study design precludes definitive attribution of these environmental factors to observed prevalence differences.

Our longitudinal observations demonstrate a consistent positive correlation between grade progression and myopia prevalence across all three geographical regions in Shaanxi Province, aligning with established epidemiological patterns in East Asian populations (8, 9). This progression likely reflects the cumulative impact of multiple risk factors:

1. Academic Intensity

Upper-grade students experience prolonged near-work duration through extended classroom hours and homework assignments, consistent with the WHO's identified threshold for myopia risk (10). Concurrently, increased access to digital devices in higher grades may exacerbate ocular strain through sustained accommodative demands.

2. Behavioral Modifications

The observed reduction in outdoor activity time among senior students corresponds with longitudinal evidence demonstrating the protective effect of daylight exposure on axial elongation (10–12). Furthermore, sleep deprivation patterns in upper grades may potentiate refractive error progression through circadian rhythm disruption, as postulated in recent pathophysiological models (13, 14).

3. Gender Disparity

The elevated myopia prevalence in female students aligns with two mechanistic pathways:

- Biological: Pubertal development stages showing differential impacts on ocular growth trajectories between genders (15, 16)
- Behavioral: Gender-specific patterns in near-work activities and outdoor exposure preferences

These findings underscore the necessity for gender-specific intervention strategies in school-based myopia control programs.

Our study revealed a distinct geographic gradient in pre-myopia prevalence across Shaanxi Province, with Southern Shaanxi demonstrating the highest detection rate, followed by Guanzhong and Northern Shaanxi. Notably, we observed a significant inverse correlation between school grade levels and pre-myopia prevalence, with male students exhibiting higher rates compared to females. These findings contrast with previous reports by Sun (17) that found no significant demographic associations, potentially reflecting regional differences in myopia progression dynamics.

These epidemiological findings reveal that primary school students in northern Shaanxi exhibit significantly higher prevalence rates of myopia and high myopia compared to other geographical regions in China (17). This distinct epidemiological pattern highlights the critical need for implementing effective strategies to decelerate myopia progression during childhood, particularly given the substantial ocular morbidity associated with high refractive errors. Notably, high myopia substantially elevates the risk of developing potentially sight-threatening ocular complications including cataract formation, retinal detachment, macular degeneration, and progressive retinal degeneration (18–20).

This investigation has several methodological limitations that warrant consideration. First, the utilization of non-cycloplegic autofocus autorefractometry for large-scale vision screening—while operationally practical for epidemiological studies—may yield systematically lower refractive error measurements compared to gold-standard cycloplegic refraction (21, 22). This methodological constraint necessitates cautious interpretation of refractive data, particularly when defining precise myopia severity thresholds. However, the protocol demonstrates superior feasibility for population-level preliminary screening given the prohibitive time requirements (average 45–60 min per subject) and logistical challenges associated with cycloplegic examinations in school-based settings. To reconcile diagnostic accuracy with operational practicality, future iterations should adopt a two-phase approach: initial mass screening through non-cycloplegic autorefraction followed by stratified random sampling for confirmatory cycloplegic refraction in representative subgroups (*n* ≥ 10% of total cohort).

Our population-based study reveals three critical epidemiological patterns of pediatric myopia in Shaanxi Province:

1. Significant regional disparities—Northern Shaanxi demonstrates a substantially higher prevalence of myopia and high myopia compared to Guanzhong and Southern Shaanxi potentially associated with differential sunlight exposure patterns and educational pressure metrics.
2. Gender-specific susceptibility—Females exhibit a increased risk of developing myopia but paradoxically demonstrate lower pre-myopia detection rates, suggesting potential biological dimorphism in accommodative responses or social-behavioral differences in near-work activities.
3. Age-dependent progression—Myopia prevalence escalates exponentially from Grade 1 to Grade 6, underscoring the critical window for intervention during the pre-myopia phase (emmetropia with ≤+0.75 D spherical equivalent).

These findings necessitate a paradigm shift in public health strategy:

Primary prevention should commence at preschool age (3–6 years), leveraging digital vision monitoring systems to establish longitudinal refractive profiles.

Secondary prevention requires standardized dynamic surveillance protocols:

Quarterly cycloplegic refractions for pre-myopes (SE: + 0.75 D to −0.50 D). Personalized interventions combining outdoor time optimization, and optical/pharmacological control for fast progressors (>0.5 D/year progression).

Tertiary prevention mandates multidisciplinary coordination between ophthalmologists, educators, and public health authorities to implement region-specific mitigation policies addressing northern Shaanxi's elevated high myopia risk.

It is important to acknowledge that refractive error assessments in this study were conducted without cycloplegia, which might lead to an overestimation of myopia prevalence, particularly among younger children with strong accommodative responses. Non-cycloplegic measurements tend to produce more negative spherical equivalent values due to residual accommodation, thereby increasing the likelihood of false-positive myopia diagnoses. While the use of automated refractometry and quality control procedures improved measurement consistency, the absence of cycloplegia remains a methodological limitation. Future large-scale screenings incorporating cycloplegic protocols, particularly in younger cohorts, are warranted to provide more accurate estimates of true refractive status.

## Data Availability

The original contributions presented in the study are included in the article/Supplementary Material, further inquiries can be directed to the corresponding author.
